# Tuberculosis in times of war and crisis: Epidemiological trends and characteristics of patients born in Ukraine, Germany, 2022

**DOI:** 10.2807/1560-7917.ES.2023.28.24.2300284

**Published:** 2023-06-15

**Authors:** Barbara Hauer, Stefan Kröger, Walter Haas, Bonita Brodhun

**Affiliations:** 1 Infectious Disease Epidemiology, Robert Koch Institute, Berlin, Germany

**Keywords:** tuberculosis, Ukraine, epidemiology, case finding, drug resistance

## Abstract

The Russian invasion of Ukraine in 2022 caused a large migration to other European countries, including Germany. This movement impacted the TB epidemiology, as Ukraine has a higher prevalence of TB and multidrug-resistant TB rates compared to Germany. Our descriptive analysis of TB surveillance data reveals important information to improve TB care in people displaced from Ukraine. We observed an expected increase in the number of TB patients born in Ukraine, which is, however, so far below WHO/Europe estimates.

Global crises and migration from countries with higher prevalence of tuberculosis (TB) and rifampicin/multidrug-resistant (RR/MDR) TB may impact epidemiology in low-incidence countries (defined as < 10 cases per 100,000 population [1]). Following the Russian invasion in early 2022, more than 1 million people arrived to Germany from Ukraine between February and December 2022 [2]. In order to better understand recent epidemiological changes in Germany and to improve TB care in displaced people from Ukraine, we analysed national surveillance data descriptively and compared numbers with World Health Organization Regional Office for Europe (WHO/Europe) estimates [3].

## Epidemiological tuberculosis situation and trend

**Figure 1:**
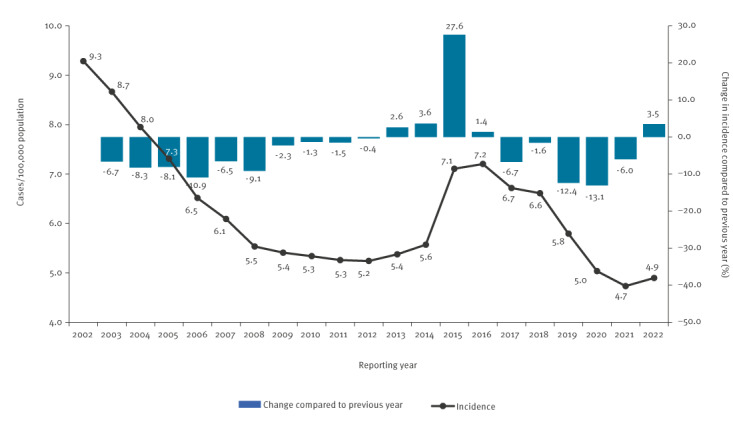
Nationwide incidence of tuberculosis, Germany, 2002–2022

In Germany, the TB incidence decline observed over the last 5 years (2017–21) came to halt in 2022, with a slight increase in incidence of 3.5% (4.9/100,000 population, Figure 1), and an almost doubled MDR-TB rate of 5.7%. We assumed this could be related to the war in Ukraine, since many people from Ukraine sought protection in Germany [2]. In 2021, Ukraine’s estimated TB incidence was 71 per 100,000 population, with high RR/MDR-TB rates (31% in new and 45% in relapse cases) [4].

Data up to 1 March 2023 were extracted and are based on population statistics of the State Statistical Offices on 31 December 2019.

## Recent notification dynamics 

Based on TB notification data obtained according to the statutory reporting obligations [5], we compared findings for 2022 with pooled data of the previous 5 years 2017–21 (cut-off date: 13 January 2023).

**Figure 2:**
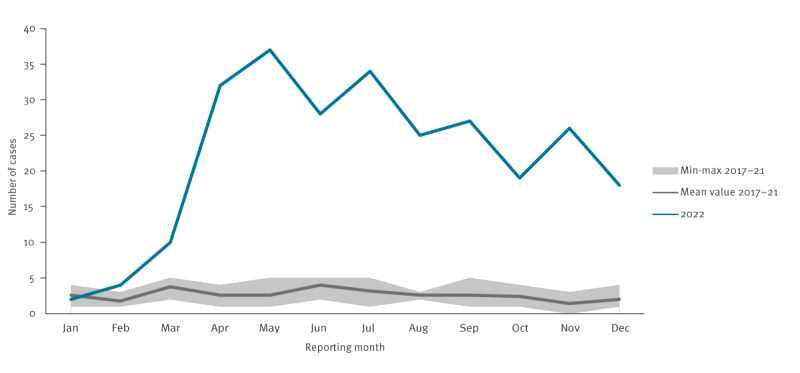
Monthly notifications of Ukrainian-born tuberculosis cases, Germany, 2022 (n = 262) compared with pooled data from 2017–2021 (n = 158)

For 262 of the 4,033 TB cases notified in 2022, Ukraine was indicated as country of birth, eight times more than in previous years (annual mean: 32 cases, range: 26–37) (Figure 2). 

Gray area shows minimum and maximum. Data up to 13 January 2023 were extracted.

## Sex and age

Sex differences were overall low (48% female; 126/261, 52% male; 135/261), but differed by age group: 25–39-year-old females were more often affected (up to 61%), and proportions were reversed in older age groups. See the Supplementary Table S1 for a summary of Ukrainian-born TB cases in Germany by age and sex. Tuberculosis epidemiology in Ukraine shows higher proportions for men [4], so this is most likely due to the different composition of the current migrant population [2].

## Site of disease and mode of case finding 

Of those with information on site of disease (n = 255), 91% (n = 232) presented with pulmonary TB, 69% (n = 175) of which were bacteriologically confirmed. Compared with previous years, the proportion of bacteriologically negative pulmonary TB was higher in 2022 (22% vs 12%; 57 cases in 2022 vs 19 cases overall for 2017–21). This may be partly due to the notification of patients already diagnosed in Ukraine, but no longer presenting with infectious TB after entry in Germany. Early TB diagnosis through active case finding measures may also play a role. In Germany, TB screening is mandatory for refugees admitted to community facilities to decrease risk of transmission and exposure of vulnerable groups (Protection against Infection Act, IfSG §36(4) [5]).

**Figure 3:**
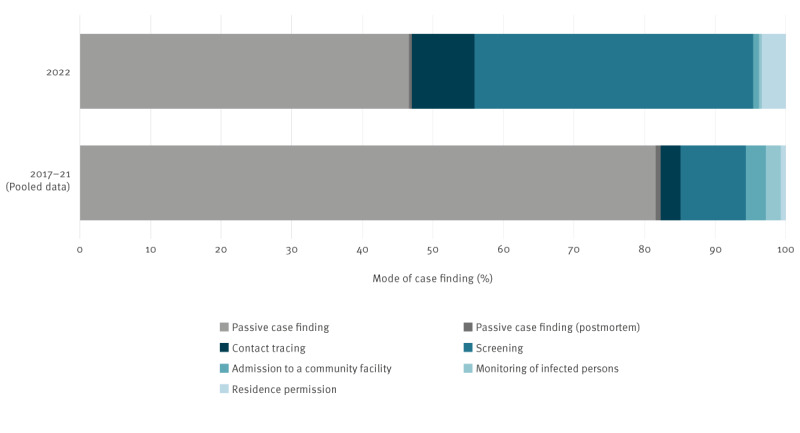
Proportion of Ukrainian-born tuberculosis cases by mode of case finding, Germany, 2022 (n = 238) compared with pooled data from 2017–2021 (n = 141)

Overall, the proportion of TB detected by active case finding measures was found to be higher in 2022 than in previous years (53%, n = 126 vs 18%, n = 25) (Figure 3). Here, TB was mainly detected through screening (n = 94, 40% of 238 cases with available information) and contact tracing (9%, n = 21).

Data up to 13 January 2023 were extracted.

## Childhood tuberculosis

Overall, 30 Ukrainian-born children (aged < 15 years) were notified with TB (13 male, 17 female); ten were aged below 5 years, 12 were aged 5–9 years, and 8 were aged 10–14 years. Of the 25 childhood TB cases with available information on site of disease, seven had extrapulmonary TB. Of the 18 pulmonary TB cases, 13 were bacteriologically negative. Most cases were detected by contact tracing (n = 14 cases), eight were detected through screening, five presented with symptoms and no information was available for three children.

## History of previous tuberculosis and drug resistance 

In Ukraine, a high proportion of TB patients have been previously treated (26% in 2021 [4]). This is also reflected by our data and puts emphasis on the need to obtain a comprehensive patient history; in 20% of the 262 patients, previous TB was recorded (n = 53), and almost two thirds of those were notified as having received TB treatment (n = 39).

The importance of early diagnosis, bacteriological confirmation and rapid drug susceptibility testing, as well as careful planning of the treatment regimen is emphasised when analysing drug resistance rates. Of the 262 Ukrainian-born TB patients, 68 were notified with MDR-TB (26%), corresponding to an MDR-TB rate of 35% in pulmonary, bacteriologically confirmed cases (62/175). Of the 62 bacteriologically confirmed MDR-TB cases, half were microscopically positive (n = 31). Every fourth MDR-TB case showed resistance against fluoroquinolone (pre-XDR-TB, n = 17), and one case fulfilled the definition [4] of extensively drug-resistant TB (XDR-TB, additional resistance against bedaquiline).

## Comparison of notified and estimated case numbers 

We compared TB case numbers (n = 262 in 2022) with the approximations provided by WHO/ Europe’s country calculator to estimate TB resources [6] (Table), based on the official number of registered displaced people from Ukraine for Germany in 2022 [2]. Tuberculosis case numbers were well below estimations (262 vs 450 cases). This applied both for adults (232 vs 387 cases) and children (30 vs 63 cases). Consequently, the number of drug-resistant TB also fell below estimations (68 MDR-TB vs 149 RR/MDR-TB cases). However, MDR- and pre-XDR-TB proportions corresponded with drug resistance rates reported for Ukraine [4].

**Table:** Comparison of tuberculosis (TB) case numbers from national surveillance data, Germany, 2022, with numbers estimated using the WHO country calculator^a^ (version from 8 September 2022) to approximate required TB resources

Cases in Germany	Displaced people from Ukraine in 2022	Female ≥ 15 years	Children 0–14 years	Male ≥ 60 years	Adults with TB	Children with TB	RR/MDR-TB among all TB	Pre-XDR-TB among RR/MDR-TB
(Estimated) numbers by calculator^b^	1,044,286	522,143	313,286	208,857	387	63	149	40
Ukraine-born TB cases notified in Germany, 2022					231	30	68	17

MDR: multidrug resistant: RR: rifampicin resistant; TB: tuberculosis.

^a^ WHO European Region, version from 8 September 2022 (for the latest version, see [6]).

^b^ The following assumptions were used: estimated TB incidence among women over 15 years (0.05%), among men over 65 years (0.06%), among children 0–14 years (0.02%), estimated RR/MDR-TB rate among pulmonary TB patients from Ukraine (33%), estimated pre-XDR-TB rate among those (27%), composition of the refugee population as women aged ≥ 15 years (50%), children aged 0–14 years (30%) and men aged ≥ 60 years (20%) based on data from host countries.

## Discussion

Thus far, information on screening results and surveillance data from people arriving from Ukraine following the Russian invasion is scarce [7-8]. Indeed, comparing available figures would not only be hampered by a huge variation in numbers of displaced people from Ukraine but also by different approaches and implementation of active case finding measures, which also vary within countries and over time. During 2022, TB case numbers in people arriving from Ukraine also seemed to be lower than anticipated in a number of other low incidence countries, e.g. in Belgium (personal communication, Wouter Arrazola de Oñate, 11 June 2023), in France [7], and in the Netherlands [8].

One possible reason for the difference between the numbers observed in Germany vs estimated case numbers could be underreporting because of under- or delayed TB diagnosis or reporting delays. For example, anecdotal evidence shows that patients already diagnosed in Ukraine sometimes arrive with anti-TB drug supplies and may therefore access healthcare with a delay and their TB may not be notified as requested. Another reason could be that the refugee population composition on which the WHO/Europe calculation is based is different or changes over time. There may also be some uncertainties in regard to the number of people from Ukraine residing in Germany at the given time [2]. It is possible that a greater impact may result from a different TB risk profile in the currently arriving people from Ukraine in Germany compared with the general Ukrainian population [9]; the population composition arriving in Germany, however, may change with an ongoing war [10,11].

Our analysis has some limitations. Because of incomplete information on year of entry, our patient cohort includes not only those who have recently migrated but also patients living in Germany even for years. Furthermore, people arriving from Ukraine are not necessarily Ukrainian-born. However, the majority of registered people arriving from Ukraine have a Ukrainian citizenship [2]. Furthermore, we did not observe any relevant epidemiological changes for other countries of birth so far. As we aimed on informing clinical management of patients originating from Ukraine, we considered these aspects to be neglectable. In addition, since we lack systematic data on the number of people screened, we were not able to calculate a case detection rate. Especially during the first months of war, the vast majority of people arriving from Ukraine were housed privately, e.g. with relatives, friends, and volunteers [2], and therefore were not obliged to undergo TB screening. This places emphasis on the need to adequately inform about TB [12-14], also offer TB screening on a voluntary basis (covered in Germany by §19 IfSG [5]) and provide barrier-free and timely access to healthcare, hereby addressing the fear of deportation, stigmatisation and potential discrimination associated with TB. Furthermore, our surveillance data do not allow us to make any conclusions on socioeconomic risk factors or coinfections, though particularly HIV-TB-coinfection is highly prevalent in Ukraine (20% in 2021 [4]).

## Conclusion

Our analysis of German notification data on TB patients born in Ukraine shows fewer numbers than expected and high drug resistance rates among these patients. These data confirm the importance of target group-oriented TB education, timely diagnosis including rapid drug susceptibility testing and (continuation of) care, provided in coordinated cooperation with all responsible partners with the necessary expertise. Active case finding measures (screening and contact tracing) substantially contributed to case detection in this group of migrants. However, as screening can only provide a snapshot and TB may still develop even years after arrival, healthcare personal should stay vigilant and ‘Think TB'. Finally, high quality and complete data are crucial to allow for a meaningful surveillance.

## Supplementary Data

123000 Supplement
