# Supplementary material for: Tuberculosis in times of war and crisis: Epidemiological trends and characteristics of patients born in Ukraine, Germany, 2022
Source: Euro Surveill. 2023 Jun 15;28(24):2300284. doi: 10.2807/1560-7917.ES.2023.28.24.2300284 (PMC10318937; doi:10.2807/1560-7917.ES.2023.28.24.2300284)
Supplement: Supplement [file 23-00284_HAUER_SUPPLEMENT.pdf]

This supplementary material is hosted by Eurosurveillance as supporting information alongside the article `Tuberculosis in times of war and crisis: Epidemiological trends and characteristics of patients born in Ukraine, Germany, 2022´ on behalf of the authors who remain responsible for the accuracy and appropriateness of the content. The same standards for ethics, copyright, attributions and permissions as for the article apply. Eurosurveillance is not responsible for the maintenance of any links or email addresses provided therein.

Supplementary TABLE S1. Notified tuberculosis cases born in Ukraine by age and sex, Germany, 2022

	**Number**		**Proportion**		**Total number**
**Age group [yrs.] / sex**	**male**	**female**	**male**	**female**	
0-14	13	17	43.3%	56.7%	30
15-19	7	6	53.8%	46.2%	13
20-24	8	7	53.3%	46.7%	15
25-29	9	14	39.1%	60.9%	23
30-39	29	36	44.6%	55.4%	65
40-49	32	27	54.2%	45.8%	59
50-59	19	9	67.9%	32.1%	28
60-69	14	5	73.7%	26.3%	19
70-79	0	2	0.0%	100.0%	2
>79	4	3	57.1%	42.9%	7
**Total (with available information)**	**135**	**126**	**51.7%**	**48.3%**	**261**
